# Singing, health and wellbeing in young children

**DOI:** 10.3389/fpsyg.2025.1595834

**Published:** 2025-08-20

**Authors:** Graham Frederick Welch, Hazel Baxter

**Affiliations:** Institute of Education, University College London, London, United Kingdom

**Keywords:** children, singing, development, health and wellbeing, exploration

## Abstract

This exploratory, pre-post study considers the impact of collective singing within inner London Primary classrooms on young children’s vocal development and sense of health and wellbeing. Data on singing and wellbeing were collected from children between the ages of five and seven before and at the conclusion of a whole class singing program. The program was led by professional singers from a charitable singing foundation who visited the school every 2 weeks over a period of 6 months (January 2024 to June 2024). Class teachers were expected to lead collective singing with their classes between the visits and the program concluded with performance in central London. Singing development was measured using the Singing Voice Development Measure (SVDM) and a revised model of vocal pitch-matching development (VPDM). Children’s perception of their health and wellbeing was assessed through the Very Short Wellbeing Questionnaire for Children (VSWQ-C), the PANAS-C measure of emotional wellbeing (modified for younger children) and focus groups at the end of the program. Results suggest that there was a significant improvement in children’s singing competency and that their perceptions of health and wellbeing were sustained across the period. However, there was no clear evidence statistically of a significant relationship between singing, health and wellbeing, primarily because, although their singing competency improved, these young children were very positive on the health and wellbeing measures throughout the focus period. Nevertheless, children in focus groups reported being very positive about the singing program and its positive impact on their health and wellbeing.

## Background

There has been a considerable growth in research studies and commentaries on the potential wider benefits of the arts —including music—on development, health and wellbeing across the lifespan (e.g., Birrell et al., 2024; Fancourt and Finn, 2019; Finn et al., 2024; Hallam and Himonides, 2022; McCrary et al., 2022; Meadows and McLennan, 2022; Welch et al., 2020). This widespread interest has coincided with an international concern over the impacts of the pandemic on general health and wellbeing, especially in children and young people living in disadvantaged circumstances (Levstek et al., 2021; Robb et al., 2023). Wellbeing as a concept in the literature is multi-faceted and includes aspects of psychological, physical and social wellbeing. Consequently, the particular focus for the current article is on children’s perceptions of their own health and wellbeing in the context of home, school, with friends and their own body (*cf.* Verrips and Vogels, 1999; Smees et al., 2020).

Several studies have explored whether it is possible for systematic engagement in creative and expressive arts activities to alleviate adverse physical and psychological conditions (e.g., Barnett and Vasiu, 2024; Finn et al., 2024; Grebosz-Haring and Thun-Hohenstein, 2024). International findings have included a reported positive correlation between the level of enjoyment of playing a musical instrument and subjective wellbeing and happiness in Chinese adults (Zhang et al., 2024), the positive impact of on-line, group music making on the wellbeing of families affected by the Zika virus in Brazil (Lisboa et al., 2024) and the beneficial impact of musical activities (singing traditional songs and musical improvisation) on the self-esteem of children forcibly displaced through violence in Colombia (Zapata and Hargreaves, 2017).

In a policy initiative, the previous UK Government commissioned an investigation into the role of the arts in improving health and wellbeing, including mental as well as physical health. The resultant report (Fancourt et al., 2020) suggested that “In relation to wellbeing, a meta-analysis of 11 RCTs [Randomized Control Trials] of music therapy for children and adolescents with psychopathology…found improvements in self-confidence, self-esteem and self-concept” (p. 9; reporting on Gold et al., 2004) and that there is “strong evidence that arts can support wellbeing in adults” (p. 10).

With regard to singing, there are a range of studies with adults that have reported benefits to mental health and wellbeing, such as the extended research by Clift and colleagues in the UK (Clift et al., 2017; Clift and Morrison, 2011; Coulton et al., 2015), including the research collated by the *Singing for Health Network*.^1^ Related research in other countries supports these UK findings, including studies in Denmark (Damsgaard and Brinkmann, 2022), Norway (Batt-Rawden and Stedje, 2020), Australia (Gridley et al., 2011) and Japan (Sakano et al., 2014). Additionally, research into the wider benefits of choral singing and health also include studies with disadvantaged adults (*cf.* Davidson and Leske, 2020), with examples reported from Australia (Dingle et al., 2013) and Canada (Bailey and Davidson, 2003, 2005), as well as a report on the efficacy of singing workshops in the UK for mental health recovery in which there was a twin emphasis on social engagement (Shakespeare and Whieldon, 2017).

In contrast to this growing body of research into the wider benefits of the arts—and music and singing in particular—on adult mental health and wellbeing, there are relatively fewer published studies involving children. A recent systematic review (Glew et al., 2021) on the effects of group singing on children and young people found thirteen studies, but only one of these included children younger than 7 years and only three studies included validated measures of wellbeing. Overall, the review authors were cautious about claims for the effectiveness of singing on psychosocial outcomes for children because of the diversity of methodological approaches and a general absence of controls. Subsequently, Davies et al. (2023) investigated the impact of a short, 2-week, 20 min-daily singing program on a class of Year 4 (8–9yo) children’s subjective wellbeing and reported a positive effect.

The possible underlying mechanisms to support such potential beneficial transfer from singing to other aspects of human functioning were explored at the beginning of the century by Thurman and Welch (2000) in their edited set of volumes *Bodymind and Voice: Foundations of Voice Education*. The “bodymind” concept derived from evidence that the human nervous, endocrine and immune systems are interlinked, drawing on the pioneering work of Candace Pert (1986, 1997) in her investigations of the psychosomatic components of illness. More recent singing research suggests that vocal self-expression is an affective process (Coutinho et al., 2019) and integral to the development of vocal identity (Welch and Preti, 2019; Davidson and Faulkner, 2019). Moreover, positive effects of singing are evidenced in biological studies, such as related to cardiovascular function (*cf.* Grape et al., 2003), and changes in the immune system through hormonal action (*cf.* Kreutz et al., 2004; see Theorell, 2019 for an overview).

Thus, it seems reasonable to hypothesize that successful singing activities with children might have a beneficial impact on their health and wellbeing. This hypothesis was the basis for an invitation from the London-based charity, the VOCES8 Foundation, to the authors to undertake an exploratory, pre-post investigation of whether a specially designed singing program for teachers and their young pupils might have a positive impact on participant children’s perceptions of health and wellbeing. Three research questions were posed in order to explore this possible link:

• Firstly, is there any evidence that the program had a positive impact on children’s assessed singing development across the period of the program, January to June 2024?
• Secondly, is there evidence of any measurable changes in the same children’s perceived health and wellbeing during this same period?
• Thirdly, is there evidence of a relationship between children’s singing development and their perceived health and wellbeing?

## Methodology

### Participants

The participants in this exploratory, pre-post study were children in Year 1 and Year 2 in a small, one form entry Primary school with a nursery in East London, situated in the London Borough of Tower Hamlets. This is a local government area to the east of the City of London and on the north bank of the River Thames. Based on the English Indices of Deprivation (2019), nationally, the local area around the school is ranked in the top 30% of greatest deprivation and has 26.6% of children living in income deprived households, being nationally ranked as 14th in the top 20 most deprived local authority districts in England. Also, Tower Hamlets has the highest proportion nationally of older people in income deprived households (43.9%). It is the worst local area in London for income deprivation overall (2024 data). Of the 205 children in the school in 2024, 61% (125 pupils) have some form of special educational need and one third (32.5%) of children are eligible for free school meals—seen as one of the main indicators of childhood deprivation. Overall, there were *N* = 58 children participating in the singing program and linked research evaluation, *n* = 29 in each year group. Two of the children had specific additional needs profiles and so only partial data were appropriate to be collected.

### Research tools

There were two main strands of quantitative research: These focused on (a) children’s singing behavior and development, and (b) their perceived health and wellbeing. With regards to (a) their singing behavior and development, each child was assessed individually against their performance of two well-known songs—*Twinkle, Twinkle Little Star* and *Happy* Birthday—using two established rating scales (Rutkowski, 1997; Welch, 1998—see Mang, 2006; Welch et al., 2012 for more detail). The resultant data from the four ratings (two per song) were combined into a “normalized singing score” (NSS) out of 100 for each child in order to enable ease of comparison between children and also to demonstrate evidence of any changes in their singing competency over time. In general, score ratings above 90 out of 100 suggest an overall tendency for the child’s singing to be in-tune and in time, with accurate lyrics and an appropriate singing register usage. Vocal behaviors rated as scoring 40 or below imply that the child is essentially still at a developmental phase of speaking the lyrics, with little sense of musical key or melodic shape. All individual singing behavior scores were agreed by the two members of the research team, drawing on a visual display of the child’s singing behavior using the software program “SING&SEE”^2^ running on a MacBook Pro.

**FIGURE 1:**
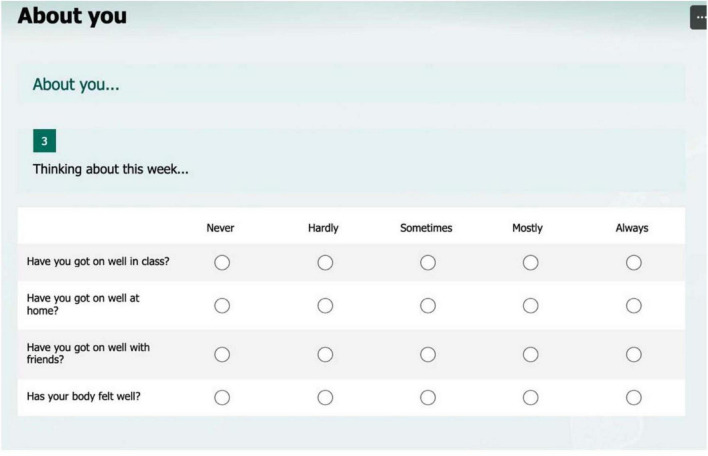
The *Very Brief Wellbeing Questionnaire for Children (VSWQ-C)* tablet presentation of statements for the assessment of children’s perceptions of key aspects of their lives, adapted from Smees et al. (2020) with permission from the American Psychological Association under license ID 1636640-1.

The assessment of (b) children’s perceived health and wellbeing was based on their responses to a set of simple statements presented on a tablet computer screen, drawing on previous research by Smees et al. (2020). Smees et al. (2020) had designed and validated two associated measures: the *Very Brief Wellbeing Questionnaire for Children (VSWQ-C)*, a health-related quality-of-life scale designed to be suitable for young children from the age of 6 years. This consists of four questions (Figure 1).

**FIGURE 2:**
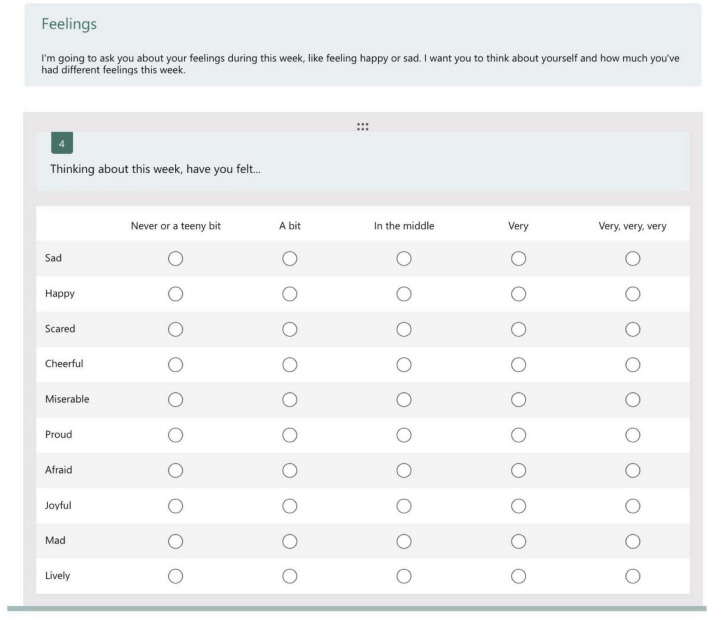
The *Definitional Positive and Negative Affect Schedule for Children (dPANAS-C)* tablet presentation of statements for the assessment of children’s perceptions of their feelings, adapted from Smees et al. (2020) with permission from the American Psychological Association under license ID 1636640-1.

Their research also adapted an existing measure of perceived emotional experiences, the *Definitional Positive and Negative Affect Schedule for Children (dPANAS-C)* which consists of 10 statements about feelings, five of which are positive and five negative (Figure 2).

Using a tablet-based approach, each statement for these two sets of measures was read out to the child by a member of the research team whilst they looked at it, and the child then selected an appropriate circle to indicate the nature of their agreement with the statement.

All responses were recorded online using the tablet interface for subsequent coding and anonymized analyses. These two sets of singing and health and wellbeing assessments were made twice, once at the beginning of the program in January 2024 and then repeated at the program’s end in June 2024.

In addition to the quantitative measures, opportunity was taken to gather qualitative data from focus groups of children who were interviewed immediately following their morning rehearsal for the public performance of the VOCES8 program in the VOCES8 Centre in the City of London in June 2024.

Ethical approval for the research was provided by UCL in January 2024 *(REC*1161: *The VOCES8 Foundation: Evaluating the impact of a singing project on the wellbeing and vocal development of children)*.

## Findings

### Quantitative data

#### Singing

**FIGURE 3:**
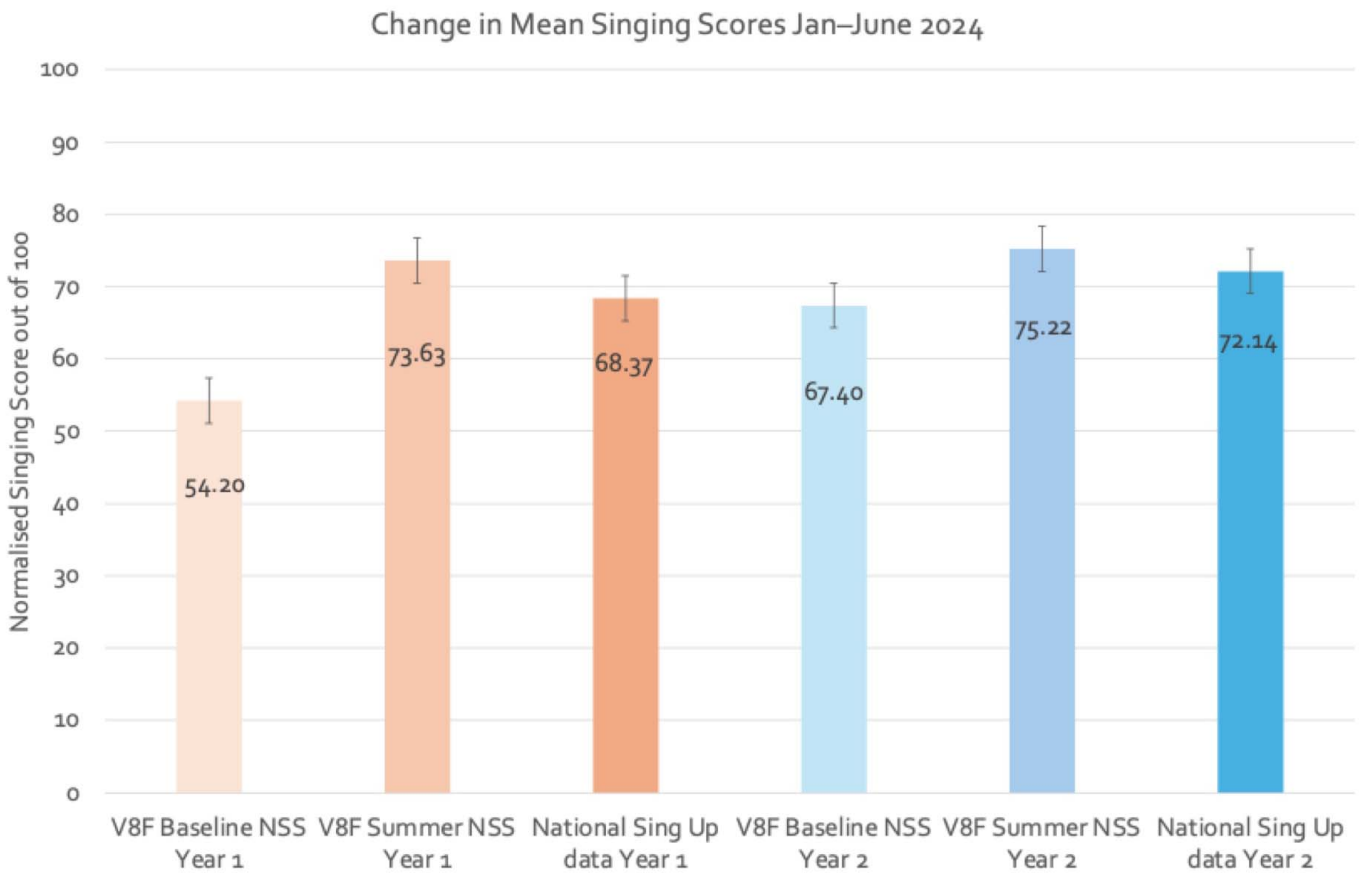
Mean normalized singing scores (NSS) for participating children in Year 1 (aged 6+y) and Year 2 (aged 7+y) for January (baseline) and June (post-program) 2024, with comparative data from the *Sing Up* national dataset for children of the same age (Year 1, *n* = 113; Year 2, *n* = 972).

Each child’s singing competency was assessed twice, namely at Baseline in January 2024 and again at the Follow-up in June 2024 at the end of the VOCES8 (V8F) program. The Normalized Singing Scores (NSS) for each Year group of children were collated and illustrated as means in Figure 3 for the two time points (January and June 2024). Means were also compared with equivalent data from UCL’s national *Sing Up* research dataset for 1,085 children of the same ages which should be treated more as a normative benchmark for children of a similar age in this particular instance rather than seen as a matched control group.

The main findings from the singing behavior analyses across the two time points are as follows:

• On average, children in both the Year 1 and Year 2 classes improved their singing competency across this 6-month period of program activity.
• Of the 56 children assessed twice, 41 improved their NSS, i.e., 73% of participants.
• In each class, the collective Summer (June) assessment data for these 56 children was *above* the mean for children of the same age who had experience of the national *Sing Up* program as recorded in our UCL national dataset (1,085 children).
• Results of a paired *t*-test for the Year 1 children indicate that there is a statistically significant large improvement between their Normalized Singing Scores (out of 100) (NSS) from Baseline (*M* = 54.2, SD = 22.2) to the Summer (*M* = 75.5, SD = 20.2), *t*(28) = 4.7, *p* < .001, *d* = .88.
• Results of the paired t-test for the Year 2 children indicated that there is a statistically significant small improvement between Baseline data (*M* = 67.4, SD = 20.1) and Summer (*M* = 75.3, SD = 18.2), *t*(28) = 2.6, *p* = .015. *d* = 0.48.

Collectively, these results indicate (a) that the VOCES8 program had a statistically significant beneficial impact on the development of participant children’s singing competency across these two school terms and (b) that the improvement compares favorably with a national dataset.

#### Health and wellbeing

With regards the data on health and wellbeing, these are reported separately below for the two scales that were used in the assessment. Firstly, the *Very Brief Wellbeing Questionnaire for Children (VSWQ-C)* data derives from children’s responses to the four-item self-report questionnaire which covers perceptions of key aspects of their lives: home life, school life, friends, and health (*cf* Smees et al., 2020).

**FIGURE 4:**
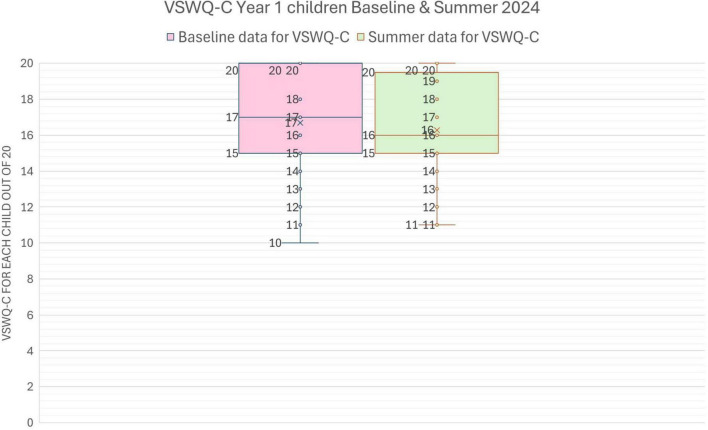
Box and whisker chart of the distribution of VSWQ-C scores for Year 1 children at Baseline (January) and Summer (June 2024). The chart illustrates the minimum, median (the middle response value), and maximum scores, with “x” shown as the mean (average).

The children in Year 1 reported relatively high wellbeing ratings at each of the two assessment points, January (Baseline) and June 2024. As can be seen from Table 1, out of a maximum score of 20 (4 questions x 5 maximum rating points, Figure 1), the mean ratings were 16.7 (Jan) and 16.3 (June), with standard deviations of 2.9 and 2.8. In other words, as a group these children were consistently very positive in their perceptions of their wellbeing (Figure 4). Results of a paired-*t* test for the whole Year 1 class indicate that there is a non-significant very small difference in *VSWQ-C* data between Baseline (*M* = 16.7, SD = 2.9) and Summer (*M* = 16.3, SD = 2.8), *t*(28) = 0.6, *p* = = 0.542.

**TABLE 1:** *VSWQ-C* Statistics for Year 1 children in January and June 2024 (maximum score = 20).

	January	June
Mean =	16.7	16.3
stdev =	2.9	2.8
1st quartile =	15	15
2nd quartile	17	16
3rd quartile	20	19
4th quartile	20	20

Within the overall set of Year 1 wellbeing responses, there were nine children with scores < 15 (the lowest 25% of scores). Over time, results of a paired *t*-test indicated that there is a significant medium difference in means between *VSWQ-C* at Baseline (*M* = 13.2, SD = 1.9) and Summer (*M* = 15.9, SD = 2.8), *t*(8) = 2.4, *p* = .046. Children with the lowest wellbeing perceptions at Baseline on this measure improved over time.

However, although there was an overall improvement in the same sub-set of children’s Normalized Singing Scores from a mean NSS of 53.1 in January to 69.8 in June, this just failed to be statistically significant [*t*(8) = 2, *p* = .085]. Thus, looking at the two measures together—singing competency and wellbeing—there is no clear evidence that changes in *VSWQ-C* wellbeing ratings for these children were associated with changes in their singing competency. This interpretation was confirmed by a comparison of change scores over time in these two measures in which the correlation was statistically non-significant (*r*_*s*_ = -0.24051, *p* (2-tailed) = 0.53).

**FIGURE 5:**
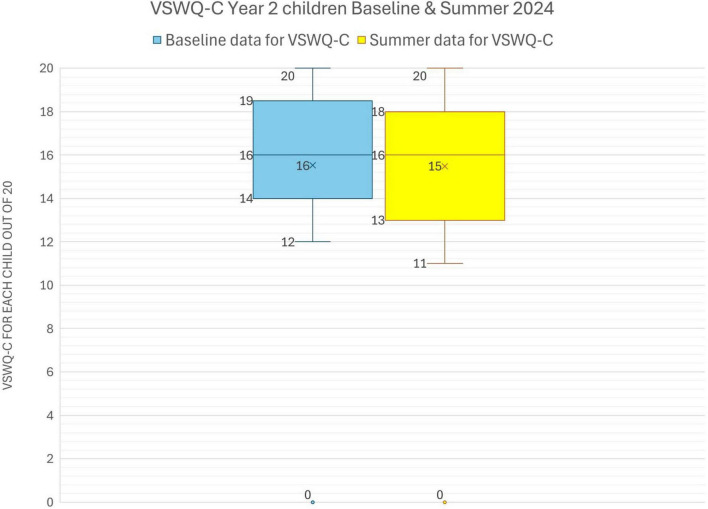
Box and whisker chart of the distribution of *VSWQ-C* scores for Year 2 children at Baseline (January) and Summer (June 2024). Chart illustrates the minimum, median (the middle value), and maximum scores, with “x” as the mean (average).

The children in Year 2 also reported relatively high wellbeing ratings at both the assessment points, January (Baseline) and June 2024. As can be seen from Table 2, out of a maximum score of 20 (4 questions x 5 maximum rating points, and Figure 1), the mean ratings were 15.5 (January) and 16.0 (June), with standard deviations of 4.0 and 2.8 (Table 2). Results of a paired *t*-test for the whole Year 2 class indicate that there is a non-significant very small difference between wellbeing measures at Baseline (*M* = 15.5, SD = 4.0) and Summer (*M* = 16, SD = 2.8), *t*(27) = 0.05, *p* = .957. The perceived wellbeing scores for the whole Year 2 class are virtually identical over time on this measure (Figure 5).

**TABLE 2:** *VSWQ-C* Statistics for Year 2 children in January and June 2024 (maximum score = 20).

	January	June
Mean =	15.5	16.0
stdev =	4.0	2.8
1st quartile =	15	15
2nd quartile	16	16
3rd quartile	18	18
4th quartile	20	20

Within the Year 2 class, there were 10 children in the lowest quartile (25%) of scores on this measure of wellbeing at Baseline. Results of a paired *t*-test for the data on these children’s feelings indicate that there is a non-significant medium difference between Baseline (*M* = 13.2, SD = 1) and in the Summer (*M* = 15.1, SD = 3.1), *t*(9) = 1.6, *p* = 0.141, i.e., any change is likely by chance.

Similarly, although the whole class improved in their singing competency over time (Figure 3), a comparison of NSS for the same sub-group of 10 Year 2 children indicated that there is a non-significant very small difference between Baseline (*M* = 71.5, SD = 20.5) and Summer (*M* = 73, SD = 20.2), *t*(9) = 0.4, *p* = 0.669.

Thus, data for these particular children’s perceived feelings and singing competency were consistent and virtually unchanged over time. A comparison of change scores over time in these two measures for this sub-group indicated that the correlation was statistically non-significant (*r*_*s*_ = 0.53563, *p* (2-tailed) = 0.11).

**FIGURE 6:**
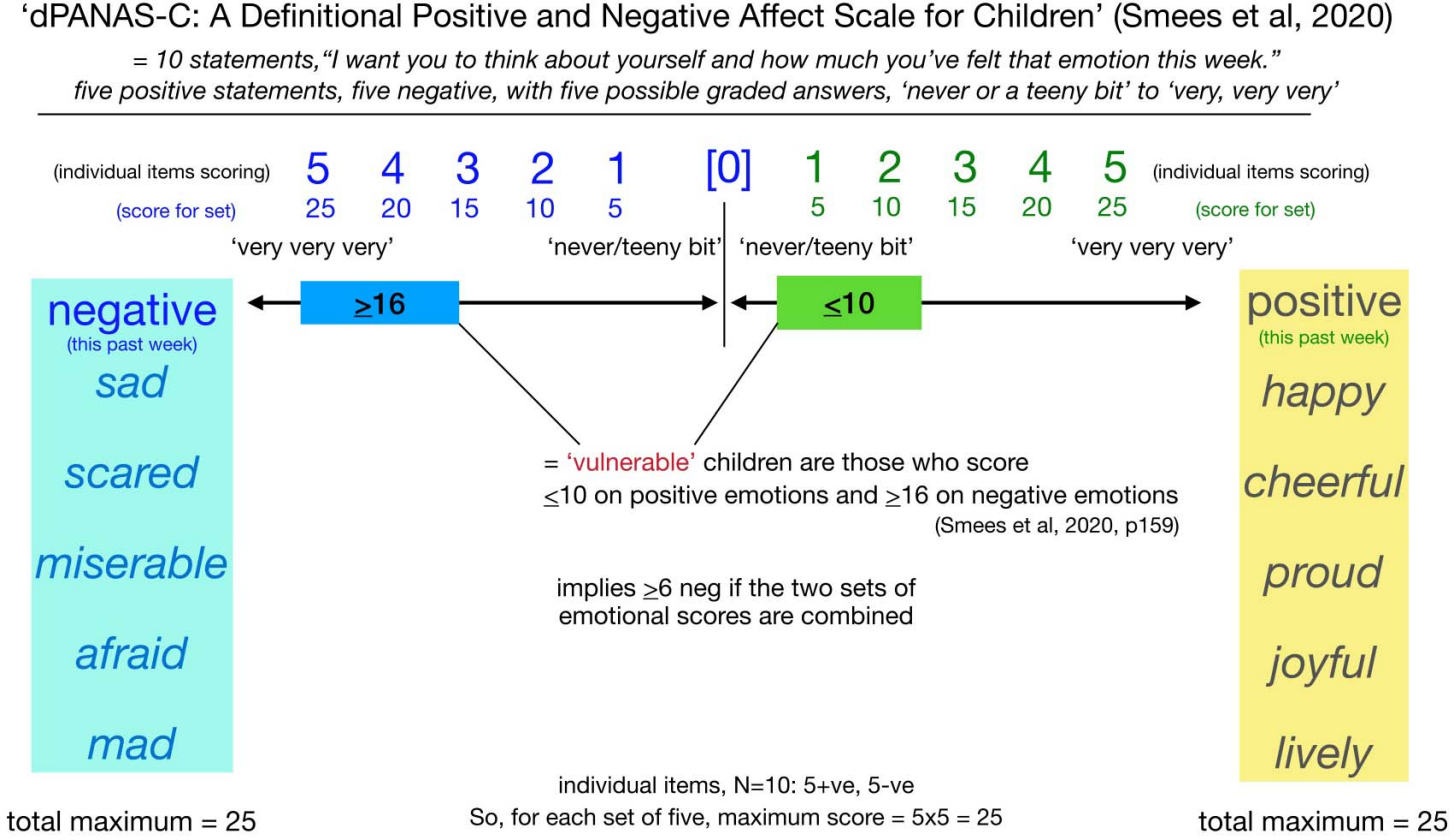
An overview of the *dPANAS-C* scale showing the positive and negative domain adjectives and how these are rated by the children on scales of 1–5 in terms of degree (“never/teeny bit” to “very, very, very”).

Data for the second of the two health and wellbeing measures, the *Definitional Positive and Negative Effect Schedule for Children (dPANAS-C)* derives from children’s responses to a 10-item self-report (Figure 2).^3^ The scale is split into two domains related to children’s feelings over the past week: *Positive Affect* relates to feelings such as happiness, pride, and activity, and *Negative Affect* relates to feelings such as sadness, fear, and anger (after Ebesutani et al., 2012). Figure 6 has been created for this report to present a composite perspective of the *dPANAS-C* scale. Children choose options in terms of their agreement with each individual item on a five-point scale, i.e., from “never/teeny bit” to “very, very, very”—in line with Smees et al. (2020) adaptation of the original version by Ebesutani et al., 2012.

In making sense of the responses, the interpretation reported by Smees et al. (2020) is that “vulnerable” children in terms of their feelings are those who choose the lower options (1 and 2) on the positive items, and higher options (4 and 5) on the negative items. In computing these responses for the inferential statistical analyses of this article, there is an assumption that each set of five positive/negative items generates a maximum rating of 5 × 5 for total agreement, thus creating a composite maximum total score of 25 for the set of 5 positive items and an equivalent composite maximum total score of 25 for the negative items for each child, with two main datasets —one for the Baseline (January 2024) and the other for the Follow-up (June 2024). Table 3 shows the negative and positive means and composite scores for Year 1 children on the *dPANAS-C* test. One child did not participate, another was absent in the Summer.

**TABLE 3:** Anonymized overview of the Year 1 *dPANAS-C* data at Baseline (January 2024) and Follow-up (June 2024) showing means, equivalent nearest categorical labels on the *dPANAS-C* scale, and the converted score out of 25 to match (Figure 6).

Baseline, January 2024						Follow-up, June 2024					
Negative feelings			Positive feelings			Negative feelings			Positive feelings		
Mean	Nearest category	Converted to a score out of 25	Mean	Nearest category	Converted to a score out of 25	Mean	Nearest category	Converted to a score out of 25	Mean	Nearest category	Converted to a score out of 25
2	A bit	10	3.2	Somewhat	16	1.6	A bit	8	3	In the middle	15
1	Never	5	5	Very, very, very	25	1.8	A bit	9	5	Very, very, very	25
2	A bit	10	3.6	Very	18	1.2	Never/teeny bit	6	4.6	Very, very, very	23
1	Never	5	5	Very, very, very	25	1	Never/teeny bit	5	4.6	Very, very, very	23
1.4	A teeny bit	7	5	Very, very, very	25	1	Never/teeny bit	5	5	Very, very, very	25
1	Never	5	4.8	Very, very, very	24	1	Never/teeny bit	5	4.8	Very, very, very	24
1	Never	5	4.6	Very, very, very	23	1.4	Teeny bit	7	4.8	Very, very, very	24
2	A bit	10	4	Very	20	1	Never/teeny bit	5	5	Very, very, very	25
1.4	A teeny bit	7	5	Very, very, very	25	1	Never/teeny bit	5	5	Very, very, very	25
1.8	A bit	9	2.6	Somewhat	13	1	Never/teeny bit	5	3	In the middle	15
1.2	A teeny bit	6	3.2	Somewhat	16	1	Never/teeny bit	5	5	Very, very, very	25
2.2	A bit	11	4.2	Very	21	1.6	A bit	8	4.4	Very	22
1.4	A teeny bit	7	4.2	Very	21	2	A bit	10	4.8	Very, very, very	24
N/a			N/A			N/A			N/A		
1.2	A teeny bit	6	5	Very, very, very	25	1.4	Teeny bit	7	4.4	Very, very, very	22
2.8	Somewhat	14	2	A bit	10	2	A bit	10	4.4	Very, very, very	22
1.2	A teeny bit	6	4.8	Very, very, very	24	1.6	A bit	8	4.2	Very	21
1.4	A teeny bit	7	5	Very, very, very	25	2.8	In the middle	14	5	Very, very, very	25
2.4	Somewhat	12	4.8	Very, very, very	24	1.6	A bit	8	4.6	Very, very, very	23
2.4	Somewhat	12	4.6	Very, very, very	23	1	Never/teeny bit	5	4.6	Very, very, very	23
2.4	Somewhat	12	4.2	Very	21	1.6	A bit	8	4.4	Very, very, very	22
2	A bit	10	4.4	Very	22	1	Never/teeny bit	5	4	Very	20
1	Never	5	5	Very, very, very	25	1	Never/teeny bit	5	3.4	In the middle	17
1.2	A teeny bit	6	5	Very, very, very	25	Missing in summer			Missing in summer		
1.8	A bit	9	4.8	Very, very, very	24	1.2	Never/teeny bit	6	4.4	Very	22
1	Never	5	4.4	Very	22	2	A bit	10	5	Very, very, very	25
2.2	A bit	11	4.8	Very, very, very	24	1.6	A bit	8	5	Very, very, very	25
2.2	A bit	11	4.2	Very	21	1.2	Never/teeny bit	6	4	Very	20
2.2	A bit	11	4	Very	20	3.4	Very	17	4.6	Very, very, very	23

**FIGURE 7:**
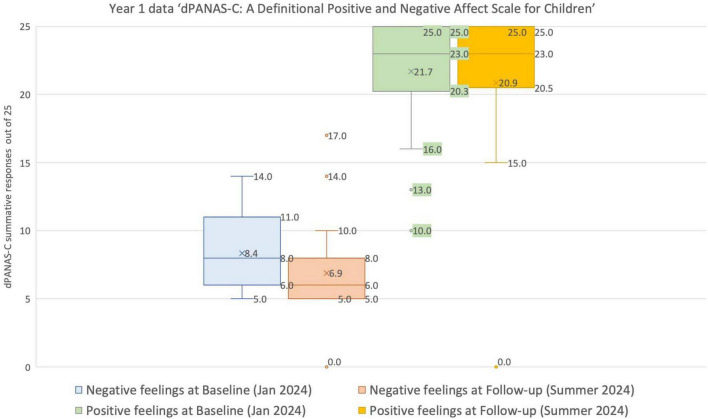
Box and whisker plot showing the range, median (most common score) and means for the *dPANAS-C* Year 1 dataset in January and June 2024.

Another way of making sense of the Year 1 children’s *dPANAS-C* data on their reported feelings over time (Table 3) is by using a Box and Whisker plot (Figure 7). Visual inspection of the plot suggests that, overall, the Year 1 children were less negative and more positive concerning their perceived feelings in the Summer. Nevertheless, these changes are non-significant statistically.^4^

The interpretation of a child’s potential ‘vulnerability’ in terms of this assessment of their perceived feelings is inferred by a composite score of ≤ 10 across the five positive items and ≥ 16 across the negative items, or a composite score of ≥ 6 if these two sets of scores are put together (see Figure 6).

With regards to the reported feelings data for these Year 1 children (Table 3):

• None of the Year 1 children had a negative score equal to or greater than 16 at Baseline.
• Only one Year 1 child had a positive score equal to or less than 10 at Baseline. However, this child was much more positive in their Summer responses.
• Subsequently, at the Follow-up assessment in June, only one Year 1 child had a negative score equal to or greater than 16. However, at the same time, this particular child was also very positive, scoring 23 out of 25 across the five positive items. The implication is that, despite researcher support, the child tended to choose the highest rating (right-hand side) responses on the tablet, irrespective of the particular feeling in question.
• Furthermore, no Year 1 child had a positive score equal to or less than 10 at the Follow-up in June.

A related *dPANAS-C* data analysis for the Year 2 children’s reported feelings demonstrates similar biases as their Year 1 peers in the distribution of scores. Table 4 shows the negative and positive means and composite scores for the Year 2 children on the *dPANAS-C* test. One Year 2 child did not participate, another was absent for the Summer assessment, and another child had joined the class since January and was first seen by the researchers in the Summer.

**TABLE 4:** Anonymized overview of the Year 2 *dpanas-C* data at baseline (January 2024) and follow-up (June 2024) showing means, equivalent nearest categorical labels on the *dPANAS-C* scale, and the converted score out of 25 to match (Figure 6).

Baseline, January 2024						Follow-up, June 2024					
Negative feelings			Positive feelings			Negative feelings			Positive feelings		
Mean	Nearest category	Converted to a score out of 25	Mean	Nearest category	Converted to a score out of 25	Mean	Nearest category	Converted to a score out of 25	Mean	Nearest category	Converted to a score out of 25
1.2	A teeny bit	6	5	Very, very, very	25	1.2	Never/teeny bit	6	4.4	Very	22
1.4	A teeny bit	7	4	Very	20	1.2	Never/teeny bit	6	2.8	In the middle	14
2.8	Somewhat	14	4.2	Very	21	1.8	A bit	9	4	Very	20
1.8	A bit	9	4.6	Very, very, very	23	1.6	A bit	8	4.4	Very	22
1	Never	5	5	Very, very, very	25	-2	A bit	-10	4	Very	20
1	Never	5	5	Very, very, very	25	1.6	A bit	8	5	Very, very, very	25
1.4	A teeny bit	7	4.2	Very	21	1.2	Never/teeny bit	6	3.6	Very	18
1.8	A bit	9	4.6	Very, very, very	23	1.8	A bit	9	3.8	Very	19
2.4	Somewhat	12	4.8	Very, very, very	24	1.4	Teeny bit	7	4.6	Very, very, very	23
1.4	A teeny bit	7	5	Very, very, very	25	Missing in summer			Missing in summer		
Not assessed						3	In the middle	15	5	Very, very, very	25
1.4	A teeny bit	7	2.2	A bit	11	2.2	A bit	11	3.2	In the middle	16
1.4	A teeny bit	7	4.6	Very, very, very	23	1	Never/teeny bit	5	3.4	In the middle	17
2.8	Somewhat	14	3.4	Somewhat	17	1.8	A bit	9	5	Very, very, very	25
2	A bit	10	5	Very, very, very	25	2.8	In the middle	14	4.2	Very	21
1	Never	5	3.4	Somewhat	17	1.4	Teeny bit	7	4.4	Very	22
1	Never	5	5	Very, very, very	25	2.8	In the middle	14	5	Very, very, very	25
2.2	A bit	11	5	Very, very, very	25	2.4	A bit	12	5	Very, very, very	25
1.8	A bit	9	4	Very	20	1	Never/teeny bit	5	4	Very	20
1.8	A bit	9	5	Very, very, very	25	1.4	Teeny bit	7	4.8	Very, very, very	24
1	Never	5	4.6	Very, very, very	23	1.6	A bit	8	2.6	In the middle	13
1	Never	5	5	Very, very, very	25	1.2	Never/teeny bit	6	5	Very, very, very	25
1	Never	5	4.4	Very	22	1	Never/teeny bit	5	4.6	Very, very, very	23
1.8	A bit	9	4.6	Very, very, very	23	1.8	A bit	9	4.8	Very, very, very	24
1	Never	5	4.6	Very, very, very	23	1	Never/teeny bit	5	5	Very, very, very	25
1.6	A teeny bit	8	4.6	Very, very, very	23	1	Never/teeny bit	5	4.6	Very, very, very	23
N/a			N/a			N/a			N/a		
2.2	A bit	11	4.2	Very	21	2.4	In the middle	12	2.8	In the middle	14
1.6	A teeny bit	8	3.8	Very	19	1	Never/teeny bit	5	4.4	Very	22
1.2	A teeny bit	6	3.2	Somewhat	16	1	Never/teeny bit	5	3.6	Very	18

**FIGURE 8:**
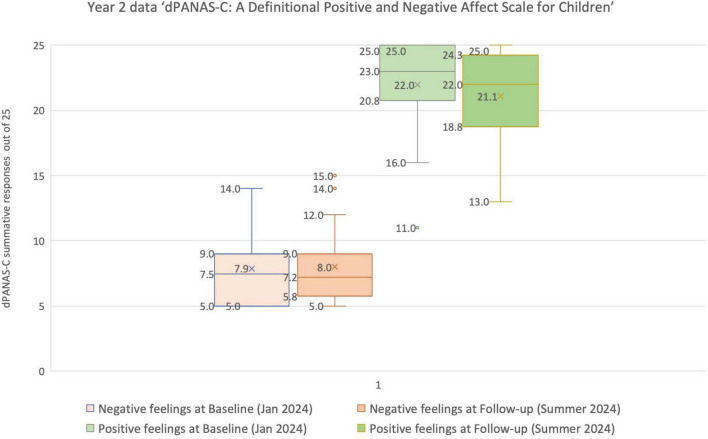
Box and whisker plot showing the range, median (most common score) and means for the *dPANAS-C* Year 2 dataset in January and June 2024.

A Box and Whisker plot of the Table 4 data reveals that, as a class, the Year 2 children tended generally to be very positive at both time points, with negative feelings being rated as relatively lowly and positive feelings clustered toward the highest ratings. Taken together, an inspection of the Year 2 feelings data against the criteria for potential “vulnerability” across these two types of presentation (Table 4; Figure 8) suggests that:

• None of the Year 2 children had negative scores ≥ 16 in January (Baseline), nor in the Summer (June, Follow-up).
• None of the Year 2 children had positive scores ≤ 10, neither in January (Baseline), nor in the Summer (June, Follow-up).

Visual inspection of the Box and Whisker plot (Figure 8) illustrates the relative consistency in Year 2 children’s responses across the two time points. This consistency was confirmed statistically; there are no significant differences between the two datasets of responses, i.e., between the ratings for negative feelings at two time points (January and June 2024), nor between the ratings for positive feelings at the same two time points.^5^

### Qualitative data

Class teachers randomly selected a small group of children from Year 1 and Year 2 to talk about their experiences of singing in the program. Two boys and one girl were selected from Year 1, and four girls and one boy were selected from Year 2. The children were informally interviewed prior to a public performance in the City of London which marked the conclusion of their singing program.

Children spoke enthusiastically about their imminent performance. Although two children said that performing made them feel shy, all children were excited at the prospect of singing to their parents.

“We’re gonna sing in front of lots of people and I’m very excited to sing because I like singing and my favorite song is Chocolate Chaud’ (Year 1 pupil)

The children reported that they had been inspired by the professional singers’ performance during that day’s morning rehearsal. When asked if they had ever heard anything like it before, one child stated,

“No, never, ever, ever. I liked it… It makes me want to sing.” (Year 1 pupil)

Children spoke entirely positively about the singing experiences that they had received in school program. Many of the children explained that they had felt excited when they knew the program singing leaders were about to visit their class.

The children demonstrated an awareness that their voices moved up and down in pitch when singing. They also highlighted the importance of warming up their voices.

“…I like changing my voice when there is a different note. Going high and low.” (Year 1 pupil)

“…they ask us to do high notes and low notes.” (Year 1 pupil)

“We warm up our voices, our brain and our bodies.” (Year 2 pupil)

“Warming up…so our voices get better for later.” (Year 2 pupil)

The children expressed their appreciation of singing collectively, the fun they had during the program singing sessions, and their enjoyment of the chosen repertoire. Many of the children volunteered their thoughts about their favorite songs.

“Everyone sings along, and it makes me feel happy because I don’t like singing by myself.” (Year 1 pupil)

“It makes me feel more confident when I’m singing in class because I’m singing with other people.” (Year 2 pupil)

“…we sing loads of fun songs.” (Year 2 pupil)

“The red, red, robin…because it’s fun!” (Year 2 pupil)

The children’s attitudes toward singing were entirely positive. The Year 2 pupils unanimously and enthusiastically declared that singing was important to them. Children also expressed their beliefs that singing is for everyone.

“You can sing while you’re doing anything. Like when you’re doing surgery if you’re a doctor, or if you’re an artist you can sing while you are painting.” (Year 2 pupil)

Children had an awareness that singing competence can improve with practice. When asked what they liked about the VOCES8 singing sessions, the response from one pupil was,

“It makes us a better singer.” (Year 2 pupil)

An awareness of the effect that singing had on their wellbeing was also very apparent from the children’s comments. All children believed that singing was a healthy activity.

“…I like singing, and sometimes when I sing it feels important to me because it makes me happy. When things are important to me, it makes me happy.” (Year 1 pupil)

“…it’s joyful.” (Year 2 pupil)

“…I sing to make me feel calm.” (Year 2 pupil)

“It helps me focus in lessons.” (Year 2 pupil)

“It makes me feel more confident.” (Year 2 pupil)

The consensus to the question, “*How does singing make you feel?”* was

“Happy!” (Year 1 pupil, and echoed by others)

## Discussion and conclusion

The overall impressions from the various types of evaluation data that were collected in this evaluation of the singing program are that:

• In answer to research question 1, the singing competency of children in both classes improved statistically over the period of the VOCES8 program. This is a positive outcome. Although the children in Year 1 made greater comparative progress statistically, this might be expected because their Year 2 peers were more advanced at the time of the Baseline assessment in January and so, perhaps, had less singing competency improvement to demonstrate.
• Also noteworthy was that the singing competency of both participant school classes improved favorably compared to children of the same age in our national *Sing Up* dataset. Each class had mean singing competency ratings that rose across this 6-month period to more than the equivalent level of children of the same age with experience of the UK Government’s *Sing Up* program at the time of our national independent evaluation across 2007–2012, with age-related singing data drawn from over 11,200 children aged 5–12 from 184 Primary schools in England.
• Analyses of the qualitative data indicated that, when asked about their singing experiences, the children reported that they very much enjoyed the singing program. It may be that the imminent public performance context for the group interviews particularly promoted the positivity of their responses. It may also be that responses reflected a bias in the focus group make-up and perhaps the collective nature of the discussion.
• With regards to possible impact of singing on the children’s health and wellbeing, the quantitative data suggest (a) that these young children aged 6 and 7 years are generally very positive about their lives, both in and out of school, and also (b) that they are more likely to report positive rather than negative feelings. Although there is some individual variation in the health and wellbeing data arising from our quantitative measures, this is not consistent. Consequently, there are no children who might be identified from this dataset as particularly and consistently emotionally “vulnerable.”

One implication is that the singing program perhaps helped to sustain this positivity as an integral component of the school culture. Children’s positivity was also evidenced in our observations of children’s behavior in class, in their willingness to sing to us individually and in small groups, in their concert rehearsal, in the final public concert performance in the City of London, and also in the enthusiastic comments about singing in the focus group data collected on the day of the performance.

It is unclear whether the overall positive bias in self-reported health and wellbeing responses at baseline and follow-up is evidence of a ceiling effect, such as produced by a limited number of response items and/or by children’s engagement with the novelty of making tablet-based choices. However, throughout the data collection, the research team took care to encourage each child to use the full range of possible responses, rather than automatically choosing an identical response to each item, and the scales were designed to require a thoughtful consideration of each statement. An alternative explanation to a ceiling effect is that our observations of the children in their normal class routines, allied to comments from their teachers and teaching assistants, as well as the findings of a recent independent Government inspection report on the quality of education in the school, suggest that, in general, these young children tend to be positive about their experiences of school and childhood.

Our national *Sing Up* data revealed a paradox of children becoming more skilled at singing as they progress through Primary school but reporting that they liked singing in school less and less (Welch et al., 2009). However, this inverse relationship was not in evidence nationally where children were in the most successful singing environments and receiving effective singing education.

With regards any possible hypothesized links between young children’s singing behavior and development and their perceived health and wellbeing, it may be that effective singing at this young age might be a contributory factor to their general sense of positivity. The absence of a statically significant relationship between the health and wellbeing data and measured singing behaviors appears to be at least partly related to the positive skewness of the former. Additionally, it may be that the chosen assessment tool for measuring individual singing competency is more sensitive to noting change; and/or it may be that any relationship between singing, health and wellbeing require a greater longitudinal perspective or older participants; and/or it may be that the experience of group singing requires a different measure, such as evidenced in several adult studies related to singing and wellbeing (e.g., Clift et al., 2017; Dingle et al., 2013; Damsgaard and Brinkmann, 2022). However, there is some evidence that Year 1 children with the lowest wellbeing perceptions at Baseline improved over time on this measure. Given the relatively small numbers of participants, this finding requires a certain caution in interpretation. Overall, the data should be seen as exploratory in nature and it would be worthwhile to undertake similar research with a larger population.

## Data Availability

The raw data supporting the conclusions of this article will be made available by the authors, without undue reservation.

## Footnotes

1
https://www.singingforhealthnetwork.co.uk/research-overview

2
https://www.singandsee.com

3 The *dPANAS-C* scale was as adapted by Smees et al. (2020) with permission from the original by Ebesutani et al. (2012), which was itself an adaptation of earlier scales with more items by Watson et al. (1988) and Laurent et al. (1999).

4 The application of a Friedman’s test for repeated measures on this Year 1 non-parametric data, in terms of its distribution, found that there was a non-significant difference over time for the negative scores [*X*^2^*_*r*_* is 1.75 (1, *N* = 28), *p* = 0.19]. Similarly, there was a non-significant difference in the positive scores [*X*^2^*_*r*_* is 0.32 (1, *N* = 28), *p* = 0.57].

5 The application of a Friedman’s test for repeated measures on this non-parametric Year 2 data, in terms of its distribution, found that there was a non-significant difference over time for the negative scores [*X*^2^*_*r*_* is 0.32 (1, *N* = 28), *p* = 0.57]. Similarly, there was a non-significant difference in the positive scores [*X*^2^*_*r*_* is 0.89 (1, *N* = 28), *p* = 0.34].
